# Structural and functional modifications of neuronal lipid rafts: implications for HIV-associated neurological disorders

**DOI:** 10.1186/s12974-026-03730-5

**Published:** 2026-02-11

**Authors:** Ying Fu, Kevin Huynh, Nigora Mukhamedova, Ben Crossett, Denise Tran, Siera Martinez, Anelia Horvath, Hong-Yin Wang, Ivan Castello-Serrano, Rosanna Ippolitto, Farhad Parhami, Peter J. Meikle, Ilya Levental, Michael Bukrinsky, Dmitri Sviridov

**Affiliations:** 1 Baker Heart and Diabetes Institute, Melbourne, VIC 3187 Australia; 2 Sydney Mass Spectrometry, The University of Sydney, Camperdown, NSW 2050 Australia; 3 McCormick Bioinformatics Core, Department of Biochemistry and Molecular Medicine, school of Medicine and Health Sciences, George Washington University, Washington, DC 20037 USA; 4 Department of Molecular Physiology and Biological Physics, University of Virginia, Charlottesville, VA 22903 USA; 5 MAX BioPharma Inc, Santa Monica, CA 90404 USA; 6 Baker Department of Cardiovascular Research, Translation and Implementation, La Trobe University, Bundoora, VIC 3086 Australia; 7 Department of Microbiology, Immunology and Tropical Diseases, George Washington University, Washington, DC 20037 USA; 8 Department of Biochemistry and Molecular Biology, Monash University, Clayton, VIC 3168 Australia

**Keywords:** HIV, Nef, Lipid rafts, Neurodegeneration, HIV-associated neurological disorder, Neurons, Extracellular vesicles

## Abstract

**Background:**

HIV-associated neurological disorders (HAND) remain a prevalent co-morbidity of HIV infection. Growing evidence implicates the HIV protein Nef in this process, as Nef increases the abundance of neuronal lipid rafts - cholesterol- and sphingolipid-rich membrane microdomains linked to neurodegeneration and neuroinflammation. Because Nef is released from infected glial cells in extracellular vesicles (exNef), we investigated whether glia-derived exNef alters the structural and functional properties of neuronal lipid rafts in a manner that could contribute to HAND pathogenesis.

**Methods:**

A comprehensive lipidomics and proteomics analysis of lipid rafts isolated from cultured human neuronal cell line SH-SY5Y, combined with assessment of biophysical and physiological properties of the rafts, was performed. We compared our findings with existing transcriptomics profiles of brain neurons from individuals diagnosed with HAND.

**Results:**

Lipidomics analysis of isolated neuronal lipid raft fractions revealed minimal impact of exNef on the raft lipid composition; physico-chemical properties of the lipid rafts were also unaffected. In contrast, proteomic analysis revealed that among raft proteins with increased abundance following exNef exposure ~25% were implicated in neurological disease pathways. Targeted protein analysis unveiled that exNef promotes the redistribution of several neurodegenerative and inflammatory proteins to the lipid rafts, resulting in increased inflammation and apoptosis alongside reduced neuronal excitability. Pharmacological disruption of raft elevation with the lipid raft antagonist Oxy210 reversed exNef-induced raft expansion and abrogated these structural and functional alterations. Importantly, changes in raft protein composition showed strong concordance with transcriptomic profiles from single-nucleus RNA sequencing (snRNA-seq) of brain from people living with HIV.

**Conclusions:**

ExNef reorganize the neuronal lipid raft proteome, enhancing the activity of neurodegenerative and inflammatory mediators. This phenomenon may potentially contribute to HAND pathogenesis.

**Supplementary Information:**

The online version contains supplementary material available at 10.1186/s12974-026-03730-5.

## Introduction

HIV-associated neurological disorders (HAND) is a frequent comorbidity of HIV infection [1]. It persists, albeit in milder forms, even when HIV replication is clinically controlled. Apart from its clinical manifestation as a neurodegenerative disease, the pathogenic mechanisms of HAND in virologically suppressed people living with HIV (PLWH) are poorly understood. HAND shares pathogenic features with other neurodegenerative disorders, including neuroinflammation [2], neuroapoptosis [2] and relocalization of intracellular β-amyloid [3]. A key mechanistic element of these common pathogenic features is impairment of cholesterol metabolism and altered abundance of lipid rafts [4]. Remarkably, the same two elements are also involved in the pathogenesis of HIV infection itself [5]. HIV is capable of modifying cholesterol metabolism and lipid rafts, not only within the infected cells but also systemically. This effect is mediated by extracellular vesicles (EVs) containing viral protein Nef (exNef).

Nef is a 27 kDa HIV-1 accessory protein synthesized early during viral replication. It is myristoylated and therefore predominantly associates with the plasma membrane in infected cells [6]. Nef can be detected in punctate membrane-proximal structures and is accessible on the surface of the cells [7, 8], a distribution pattern that is compatible with localization to lipid rafts. Nef is also present in the cytoplasm [7] and is released from infected cells within EVs [3, 9]. While Nef is dispensable for viral replication, its absence markedly attenuates HIV pathogenicity, and Nef-deficient infections rarely progress to AIDS [10]. Nef interferes with cellular trafficking of multiple host proteins, including immune receptors such as CD4, CD8, CD28, as well as the restriction factors SERINC3/5 and the antigen-presenting molecule MHC-1, reducing their surface expression and thereby impairing immune defenses and promoting viral replication [11]. Nef also elevates the abundance of lipid rafts [12], a location of most immune receptors as well as the site of amyloidogenic proteins aggregation. The mechanism by which Nef modifies lipid rafts involves reduction of the abundance of ABCA1 [13], a key regulator of cholesterol efflux that also plays a role in β-actin polymerization and cytoskeletal assembly/disassembly [14, 15]. Both ABCA1- and β-actin-dependent pathways are critical for regulating the abundance of lipid rafts [15]. Remarkably, Nef elevates the abundance of lipid rafts not only in infected cells, but also in bystander cells, via Nef-containing EVs (exNef) secreted by the infected cells into the bloodstream [3, 9] and taken up by wide variety of cells, including neurons. We recently demonstrated that exNef cause elevation of the abundance of lipid rafts, redistribution of amyloid precursor protein, and functional impairment in neurons in vitro [8] and in individuals diagnosed with HAND [16]. Based on these findings, we hypothesized that elevation of the abundance of lipid rafts by exNef might contribute to the pathogenic mechanisms of HAND [17]. It remains unclear whether changes in the lipid raft abundance are accompanied by alterations in their structure and functional properties. In this study, we examined how exNef influence the lipid and protein composition, as well as the stability of lipid rafts in human neuronal cell line. We further assessed how these changes affect neuronal function, in ways that may be relevant to mechanisms proposed in HAND.

## Methods

### HTHU microglial cells culture and Nef transfection

The immortalized human microglia cells, clone C20 [18], also known as HTHU cells (Highly Transformed Human microglial cells) [19], were a generous donation from Dr. Yoelvis García Mesa (CWRU, Cleveland, OH, USA). Cells were maintained in DMEM/F12 medium (Invitrogen) supplemented with 10% FBS, 0.1 mmol/L nonessential amino acids and 1% antimycotic/ penicillin/streptomycin (Thermo Fisher Scientific) at 5% CO_2_ in an incubator at 37 °C. The cells were split when reaching 80–90% confluence and used at passage 3. HTHU cells were transfected with GFP- or Nef-expressing plasmids using Lipofectamine 3000 (Invitrogen) following manufacturer’s instructions. Transfected cells were grown in DMEM/F12 medium containing 10% of exosome-depleted FBS for 2–3 days at 37 °C in a CO_2_ incubator. To assess the expressed GFP and Nef proteins level, cells were harvested into RIPA buffer, processed on SDS-PAGE, and analyzed with anti-Nef or anti-GFP antibodies by Western blotting.

### Extracellular vesicle isolation and purification

Nef-containing EVs (exNef) were isolated from cell culture medium of HTHU microglial cells transfected with Nef plasmid. Medium containing EVs was collected and cleared from cells and debris by centrifugation at 2,000 x g for 20 min. The medium was then combined with an equal volume of 16% Polyethylene glycol 6000 (Sigma Aldrich) in 1 M NaCl solution, incubated with agitation overnight at 4 °C, and centrifuged at 4,750 x g for 80 min. The pellet was resuspended in ice-cold PBS and centrifuged at 100,000 x g for 90 min. The purity and size distribution of the EV particles were analysed with NanoSight NS300 (ATA Scientific Instruments). Monoclonal anti-Nef or polyclonal anti-GFP antibodies were used to detect the presence of Nef or GFP in the EVs by Western blotting. Nef concentration in EVs was standardized to a known recombinant Nef (rNef) concentration. Typically, ~1 ng of Nef was detected per 1.6 × 10^8^ particles and, unless indicated otherwise, was added to cells at a concentration of 1.6 × 10^8^ particles/ml (1 ng/ml of Nef). The same number of exGFP particles was added to control samples.

### SH-SY5Y cells culture and differentiation

Human neuroblastoma SH-SY5Y cells (ATCC, Manassas, VA, USA) were cultured in DMEM/F12 medium (Invitrogen) supplemented with 10% FBS, 0.1 mM non-essential amino acids, and 1% antimycotic/penicillin/streptomycin (Thermo Fisher Scientific). Cells were maintained in T75 flasks until reaching 70–80% confluence. Cells were lifted with Accutase cell detachment solution (Sigma-Aldrich) and split at density of 3 × 10^4^ cells/cm^2^. The 15th -22nd passage of the cells was used in majority of experiments and cells were discarded after 24th passage. The cells were differentiated as described previously [20]. Briefly, SH-SY5Y cells were seeded onto collagen-coated plates or dishes in complete medium supplemented with 13 µmol/L retinoic acid (Sigma-Aldrich) for 4 days. The cells were subsequently maintained in serum-free DMEM/F12 medium containing 5 ng/ml brain-derived neurotrophic factor (BDNF, Abcam) for 3–5 days. Differentiation of the cells was assessed visually by morphologic changes and spread of neurites as well by staining for MAP2 as described previously [8].

### Lipid raft isolation

Lipid rafts were isolated as described previously [8, 21]. Briefly, cultured cells were washed and collected in cold PBS followed by a hypo-osmotic shock in 5 mM Tris-HCl buffer (pH 7.5) containing complete protease/phosphatase inhibitors. Membrane pellets were collected by ultracentrifugation at 435,000 x g for 90 min at 4 °C in a TLA 120.2 rotor. Equal amounts of membrane proteins were applied onto OptiPrep linear gradient (Sigma Aldrich). After two ultracentrifugations, fractions were collected from the top and processed for radioactivity counting (where 75 kBq/ml of ^3^H-cholesterol was added during retinoic acid differentiation stage) and/or flotillin-1 detection by Western blotting. Combined raft-enriched fractions were used in the subsequent experiments.

### Isolation of giant plasma membrane vesicles (GPMV)

Cell membranes were stained with 5 µg/ml of FAST-DiO (Invitrogen), a green fluorescent lipid dye that strongly partitions to disordered/non-raft phases [22, 23]. Following staining, GPMVs were isolated from undifferentiated THP-1 cells as previously described [24]. Briefly, GPMV formation was induced by 25 mM paraformaldehyde and 2 mM dithiothreitol in hypotonic buffer containing 100 mM NaCl, 10 mM HEPES, and 2 mM CaCl_2_, pH 7.4. To measure phase separation temperature, GPMVs were observed on an inverted epifluorescence microscope (Nikon) at various temperatures and the fraction of vesicles showing macroscopic phase separation was manually quantified. The temperature-induced changes in fraction of phase separated vesicles was then fitted to a sigmoidal curve and the calculated temperature at which 50% of vesicles were phase separated was called the miscibility transition temperature (T_misc_) [25].

### Lipid packing measurements by Di4 FLIM

Lipid packing in live cell plasma membranes or GPMVs was measured using a fluorescent reporter Di4 (Di-4-ANEPPDHQ) whose fluorescence emission lifetime is highly dependent on lipid packing [26], as previously described [27]. Outer leaflets of cultured cells were stained by incubating cells at 4°C for 8 min with Di4 at 1 µg/ml in staining buffer (10 mM HEPES, pH = 7.4, 150 mM NaCl, 2 mM CaCl_2_). Cells were washed twice in the same buffer at ambient temperature before imaging in phenol red-free MEM. For FLIM, Di4 emission was collected at > 560 nm with the instrument response function (IRF) determined with a saturated erythrosine B. Di4 images were acquired using 20 MHz pulse frequency. The photon count rate was kept under 10% of the pulse rate by adjusting a manual shutter, and enough frames were acquired to obtain at least 10^4^ photons cumulative signal intensity. The fluorescence decay curves were fitted to a bi-exponential re-convolution function adjusted to the IRF and the average lifetime was calculated and represented in the FLIM images as τ_Di4_. GPMVs were imaged at 10 °C to observe phase separation.

### Cholesterol efflux

Cholesterol efflux was measured as described previously [8]. In brief, cells were labelled with [^3^H]cholesterol (75 kBq/ml, for 48 h), and incubated with human apolipoprotein A-I (30 µg/ml for 2 h at 37˚C). The efflux was calculated as a proportion of radioactivity moved from cells to medium.

### Western blotting

Cultured cells were lysed in RIPA buffer supplemented with protease/phosphatase inhibitor mini tablets (Roche Diagnostics GmbH, Germany). The lysates were then separated on a SDS-PAGE, transferred to PVDF membrane, followed by blocking in 4% skim milk. Proteins were probed with different antibodies. Staining was detected using Lighting ECL (PerkinElmer). The images were taken using Syngene GBox image developer, quantitated with GeneTools (Syngene).

Antibodies used in the Western blotting experiments were as following. Rabbit monoclonal anti-NMDAR1 antibody (#A11699), anti-AMPA antibody (GluR2/GRIA2, #A11316), rabbit polyclonal anti-nAChR3 antibody (# A1674) and anti-APP (#A3161) were from ABClonal. Rabbit monoclonal anti-caspase-8 antibody (#4790), anti-TNF-R1 antibody (#3736), rabbit polyclonal anti-α-synuclein (#2628) were from Cell Signalling Technologies. Mouse monoclonal anti-ABCA1 antibody (#ab18180), anti-sodium potassium ATPase α-1 antibody (#ab7671), and polyclonal anti-flotillin-1 antibody (#ab50671) were from Abcam. Rabbit polyclonal anti-TLR4 antibody (#342345) was from USBiologicals. Mouse monoclonal anti-ERK1/2 antibody (#05-1152) was from Millipore, anti-phosphorylated ERK1/2 antibody (#05-481) was from Upstate/Merck. Monoclonal anti-β-actin antibody (# A1978) was from Sigma Aldrich. Mouse monoclonal anti-Nef antibody (#3689) was from the NIH AIDS Reagent Program (NIAID, NIH). Mouse monoclonal anti-Alix antibody (#NB100-65678) was from Novex Biologicals. Monoclonal anti-GAPDH antibody (#CB1001) was from Millipore. Secondary anti-rabbit IgG horseradish peroxidase conjugates (#7074) and anti-mouse IgG horseradish peroxidase conjugates (#7076) were from Cell Signalling Technologies.

### Lipidomics

SH-SY5Y cells seeded in T75 flasks were differentiated and treated with exNef or exGFP and cells were harvested into ice-cold PBS. Lipid rafts were isolated as described above. Fractions containing lipid rafts were combined according to the density of flotillin-1 presence detected by Western blotting.

Lipidomic analysis was performed as described previously [21]. Combined lipid raft fractions were sonicated on ice; lipids were extracted using a modified single phase 1:1 butanol: methanol method. In brief, samples were lyophilized and mixed with 50 µl of methanol, 50 µl of butanol and 10 µl of water. Samples were sonicated for 1 h at 25 °C before centrifugation (13,000 x g) for 10 min. The supernatant was carefully aspirated, avoiding the Opti prep precipitate, into mass spectrometry glass vials with inserts.

Lipids were separated under the following chromatographic conditions using solvent A (50% water, 30% acetonitrile and 20% isopropanol with 10 mM ammonium formate and 5 µM medronic acid) and solvent B (1% water, 9% acetonitrile, 90% isopropanol with 10 mM ammonium formate). The starting concentration of B was 15%, going to 50% at 2.5 min, 57% at 2.6 min, 70% at 9 min, 93% at 9.1 min, 96% at 11 min, 100% at 11.1 min and holding until 12 min before going down to 15% at 12.2 min and re-equilibrating at 15% B until 16 min.

Each sample was run twice, under two separate mass spectrometry conditions. Condition 1: temperature, 280 °C; gas flow rate, 17 L/min; nebulizer, 20 psi; sheath gas temperature, 400 °C; capillary voltage, 3,500 V and sheath gas flow, 12 L/min. Condition 2: temperature, 150 °C; gas flow rate, 17 L/min; nebulizer, 20 psi; sheath gas temperature, 200 °C; capillary voltage, 3,500 V and sheath gas flow, 12 L/min.

Relative lipid concentrations for each fraction were calculated by relating the peak area of each species to the peak area of the corresponding internal standard.

### Proteomics

Samples were prepared using S-Trap micro MS sample preparation kit (ProtiFi, NY, USA) which solubilises, reduces, alkylates and cleans up the proteins prior to trypsin digestion at 47 °C for 2 h. The peptides were quantified using Qubit Assay (Thermo Scientific, MA, USA) and then desalted by solid-phase extraction using 1 cc HLB cartridges (Waters, MA, USA). Peptide mixtures resuspended in 3% (v/v) ACN/0.1% (v/v) FA and 300 ng of peptides were separated by nano-LC using an Ultimate 3000 UHPLC and autosampler system (Dionex, Amsterdam, Netherlands) by direct injection onto a 30-cm×70-µm C18 fused-silica analytical column with a 10-µm pulled tip, coupled online to an electrospray ionization source. Reverse-phase mobile buffers were composed of A: 0.1% (v/v) FA (Thermo Scientific, MA, US, # 34851-4), and B: 80% (v/v) ACN (Thermo OPTIMA LC–MS grade, Cat No. 34851-4)/0.1% FA. Peptides were eluted using a linear gradient of 5% B to 42% B across 60 min with a constant flow rate of 250 mL/min. Peptides were ionized by electrospray ionization at 2.3 kV. Tandem mass spectrometry analysis was performed using a Q-Exactive Fusion Eclipse mass spectrometer (Thermo Fisher) with 27% normalized higher-energy collisional dissociation for fragmentation. Spectra were attained in a data-independent acquisition using 20 variable isolation windows. Data files were analysed using Spectronaut (version 19.0, from Biognosys, Schlieren, Switzerland). The database provided to the search engine for identification contained the UniProt human database downloaded on Jan 13th 2023. The false discovery rate was set to 1% of precursor ions. Both remove likely interferences and match between runs were enabled. Trypsin was set as the digestion enzyme with a maximum of 2 missed cleavages. Carbamidomethylation of Cys was set as a fixed modification, and oxidation of Met was set as variable modifications.

The mass spectrometry proteomics data have been deposited to the ProteomeXchange Consortium via the PRIDE partner repository with the dataset identifier PXD065713.

### TUNEL assay

SH-SY5Y cells were seeded at 2.5 × 10^4^ cells per well in 96-well plates and differentiated as described above. exGFP and exNef treatment was carried out during the BDNF differentiation stage in serum-free medium, when indicated supplemented with 10 ng/ml TNFα for 72 h. Post 72-h TNFα treatment, cells were treated with 1.25 mM methyl-β cyclodextrin (Sigma-Aldrich) for 15 min at 37 °C. The cells were then rinsed with phosphate-buffered saline pH 7.4 (PBS) and fixed with 3.7% neutral-buffered formaldehyde (Fronine Laboratory Supplies, Australia) for 7 min and post fixed in 100% cold methanol for 10 min. Fixed cells were permeabilized with 0.1% Triton X-100 in PBS for 10 min at room temperature, followed by two times wash with PBS. The fixed and permeabilized cells were then subjected to the TUNEL assay according to the manufacturer’s instructions (HT Titer TACS™ Apoptosis Detection Kit R&D Systems, Inc., USA). Apoptotic cells were expressed as percentage of TACS positive control. The experiments were repeated several times. The TACS nuclease positive and unlabelled controls were included in each TUNEL assay.

### MTT assay

SH-SY5Y cells cultured in 96-well plates, incubated with exGFP or exNef, treated with TNFα and/or mβCD as described above. Then, 0.5 mM MTT solution (Sigma Aldrich) was added to cell wells, cells were incubated up to 2.5 h until purple colour appeared. The reaction was terminated by adding equal volume of DMSO for 15 min. The absorbance at 570 nm was measured in a microplate reader.

### Nicotinic acetylcholine receptors (nAChR) assay

SH-SY5Y cells were seeded at 2.5 × 10^4^ cells per well in 96-well plates and differentiated as described above. Cells were treated with exGFP or exNef during the BDNF differentiation stage in phenol-red-free and serum-free DMEM/F12 for 48 h. Then, cells were loaded with 100 µl of 5 µM cell-permeable Fura-2 acetoxymethyl ester (Fura-2 AM, Thermo Fisher Scientific) in Krebs-Ringer Bicarbonate Buffer (KRBB) buffer (110.8 mM, NaCl, 4.87 mM KCl, 1.21 mM MgSO_4_, 1.22 mM, KH_2_PO_4_, 25.7 mM NaHCO_3_, 10 mM HEPES and 1 mM Probenecid (Sigma-Aldrich)), and incubated in the dark for 45 min at 37 °C. After incubation, cells were washed with the KRBB solution, treated with 100 µl of 50 µM nicotine (Sigma-Aldrich) or vehicle per well for 15 min. Changes in fluorescence (excitation 340 and 380 nm, emission 520 nm) were measured using a FLUOstar Optima Microplate Reader. Relative fluorescence was calculated as a ratio of fluorescence intensity at 340 to 380 nm. ΔR denotes the difference in fluorescence ratio between nicotine-treated and non-treated cells.

### Single-nuclei RNA sequencing analysis of human brain data

Post-mortem flash-frozen brain samples of middle temporal gyrus of PLWH (two diagnosed with HIV-associated dementia (HAD), a severe form of HAND, and two with Asymptomatic Neurocognitive Impairment (ANI), a mild HAND form, were obtained from National NeuroAIDS Tissue Consortium. In addition, comparative single-nucleus RNA-seq data from four age-matched uninfected individuals were obtained from published reports, which can be accessed from GEO (SRR19918318, SRR19918319, SRR19918320, SRR19918322, SRR19918323, SRR19918324, SRR19918325, SRR19918327), and these datasets were processed and integrated following the same computational pipeline to ensure consistency across cohorts.

The Seurat software package (v5.1.0) and Signac (v 1.14.0) in R and their standard workflows were used to perform quality control, clustering, integration, and differential gene expression analysis. The data were filtered by the following parameters: 200< nFeature_RNA < 9500, percent.mt < 5, nCount_RNA < 60,000, nucleosome_signal < 2, and 2 < TSS.enrichment < 9. The samples were merged, scaled/normalized (SCTransform), integrated (CCAIntegration), dimensionally reduced (PCA and UMAP) and cell typed using the R package ScType and a modified gene list for the brain tissue type (v2021).

Differential gene expression analysis and GO and KEGG GSEA were performed using the Wilcoxon Test and the gseGO and gseKEGG functions from the R package clusterProfiler v4.12.6 using the ranked average log2FC differential gene expression. Results were filtered to include only adjusted p-values of less than or equal to 0.05. Figures were created using R packages Seurat and ggplot2 (v3.5.1) and python’s matplotlib.

### Statistics

All data are shown as mean ± SEM, unless stated otherwise. Statistical significance was determined using 2-way ANOVA with Shapiro-Wilk test for normality, or Mann-Whitney test, with P value < 0.05 defining significant difference. GraphPad Prism 10 was used to examine the data for statistical significance between groups.

## Results

### EVs from microglial cells

Neurological co-morbidities of HIV infection persist even during successful antiretroviral therapy (ART), when no viral particles are detectable in the blood and only a small number of HIV-infected cells remain within viral reservoirs [28]. One possible explanation for this phenomenon involves “bystander” effects, in which individual HIV-derived proteins, often released in EVs from the few remaining infected cells, affect multiple cells, including those that cannot themselves be infected by HIV [29, 30], such as neurons [9, 31].

**Fig. 1:**
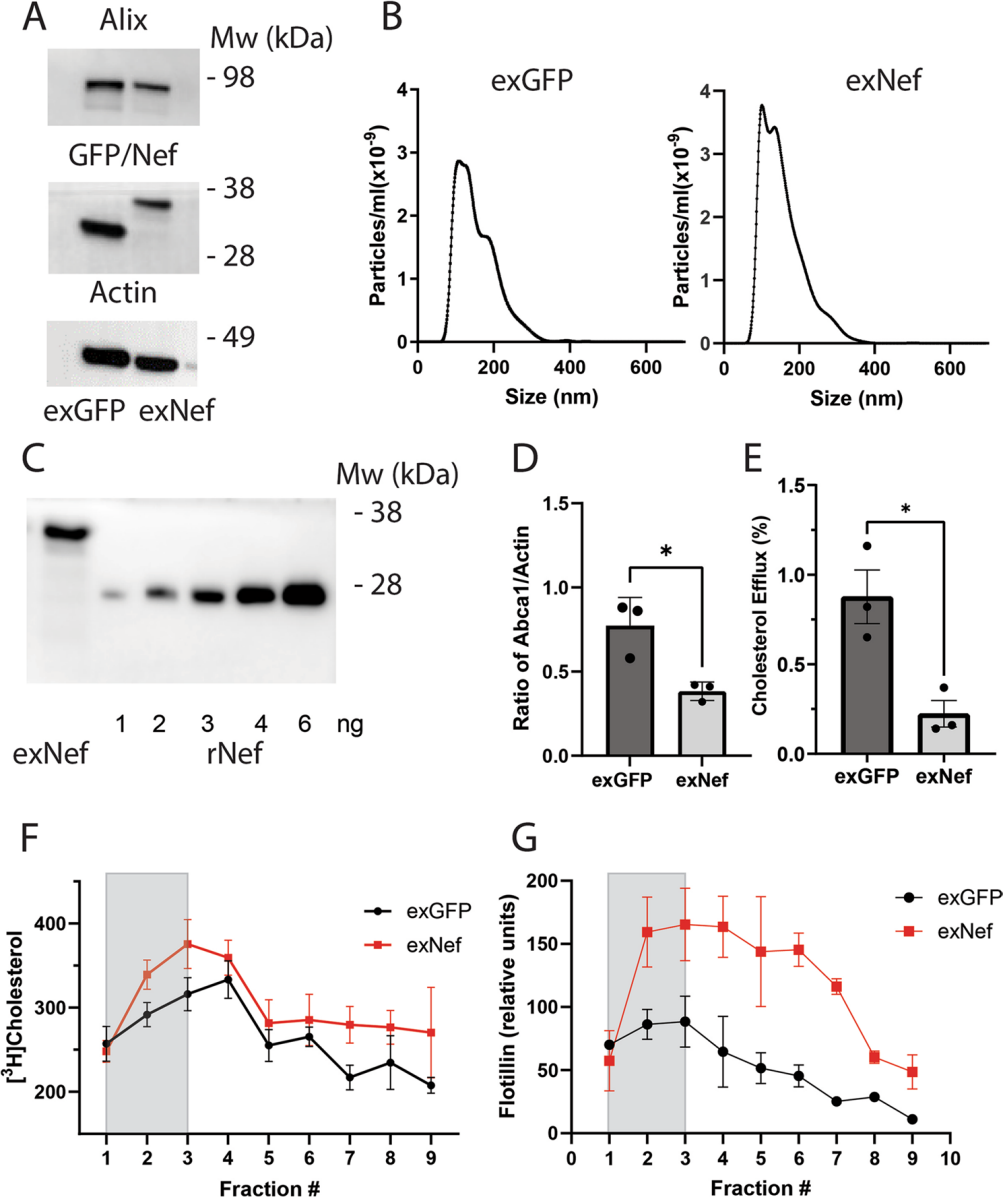
Characterization of EVs produced in human microglia cells HTHU. **A ** The presence of Nef, GFP and Alix in isolated EVs analysed by Western blot; **B** Size distribution of EVs; **C** Calibration Western blot for assessing Nef content in exNef; **D** The effect of exNef (1.6 × 10^8^ particles 48 h) on the abundance of ABCA1 in SH-SY5Y cells; Means ± SEM; *p < 0.05 (2-way ANOVA, *n* = 3, biological replicates); **E **The effect of exNef on cholesterol efflux from SH-SY5Y cells to apolipoprotein A-I (30 µg/ml 2 h); (Means ± SEM; *p < 0.05 (2-way ANOVA, *n* = 3, biological replicates); **F **The effect of exNef on the abundance of lipid rafts quantified by the incorporation of [^3^H] cholesterol ); (Means ± SD, *p < 0.05, Mann-Whitney test, *n* = 3, biological replicates); **G** The effect of exNef on the abundance of lipid rafts quantified by the abundance of flotillin-1 (Means ± SD, *p < 0.05, Mann-Whitney test, n = 3, biological replicates). Shaded areas are defined as lipid raft-enriched fractions

We recently reported that EVs containing HIV-1 protein Nef modify lipid rafts in neuronal cells [8]. An important limitation of that study is that the EVs were produced in HEK293 cells, whereas in the brain of PLWH Nef-containing EVs most likely originate from infected microglial cells, the primary HIV reservoir in the brain [32]. Since the origin of the EVs is important for their functional activity, in this study we employed EVs produced by Highly Transformed Human Microglial (HTHU) cells. The isolation of EVs secreted by HTHU cells expressing either Nef or green fluorescent protein (GFP, control EVs) is described in the Methods section; they are termed exNef and exGFP, respectively. Similar to EVs produced in HEK293 cells, HTHU-produced exGFP and exNef contained, respectively, GFP and Nef and were positive for the EV marker Alix (Fig. 1A). The predominant size range of EVs was 120–150 nm (Fig. 1B). The concentration of the Nef protein in the exNef was quantified by Western blot by comparing to signal from recombinant Nef (Fig. 1C), it was typically ~1 ng of Nef per 1.6 × 10^8^ particles.

To assess the functional activity of exNef produced in HTHU cells, we evaluated its ability to reproduce two key cellular effects described for exNef produced by HEK293 cells: downregulation of the cholesterol transporter ABCA1 and inhibition of cholesterol efflux [12, 15]. We demonstrated that exNef produced by HTHU cells effectively reduced the abundance of ABCA1 (Fig. 1D) and reduced cholesterol efflux to apolipoprotein A-I (Fig. 1E) from SH-SY5Y cells, similar to the effects observed with exNef produced in HEK293 cells [8].

To assess the effect of exNef on lipid rafts, we isolated raft-enriched fraction of the plasma membrane from cells treated with exNef or exGFP using a previously described procedure [15, 21]. SH-SY5Y cells were labelled with [^3^H]cholesterol and equal amounts of isolated plasma membranes were fractionated by density gradient centrifugation. Profiles of [^3^H]cholesterol and flotillin-1 distribution along the density gradient are shown in Figs. 1F, G. Lightest fractions consistently containing elevated amounts of [^3^H]cholesterol and enriched in flotillin-1 (typically, fractions 1–3, shaded in Figs. 1F, G) were defined as raft-enriched fractions. These fractions were termed “lipid rafts”, however, it is important to recognize that they are not “pure” lipid rafts, but a raft-enriched fraction of the plasma membrane. Treatment of cells with exNef for 48 h resulted in a statistically significant (*p* < 0.02, t-test, *n* = 3) elevation in the abundance of lipid rafts.

Treatment with GFP-containing EVs was used as a control throughout this study. Because the lipid constituents of EVs may potentially influence lipid raft composition, it was essential to control the effects of exNef using EVs carrying an unrelated protein of similar size that is not membrane-associated, thereby avoiding potential confounding effects on raft structure. We have previously demonstrated that the effects of exNef closely mirror the effects of myristoylated recombinant Nef, although the latter requires higher concentrations, supporting the conclusion that the observed changes are attributable to Nef itself rather than to other EV components [8, 33].

### Lipid composition of neuronal lipid rafts

**Fig. 2:**
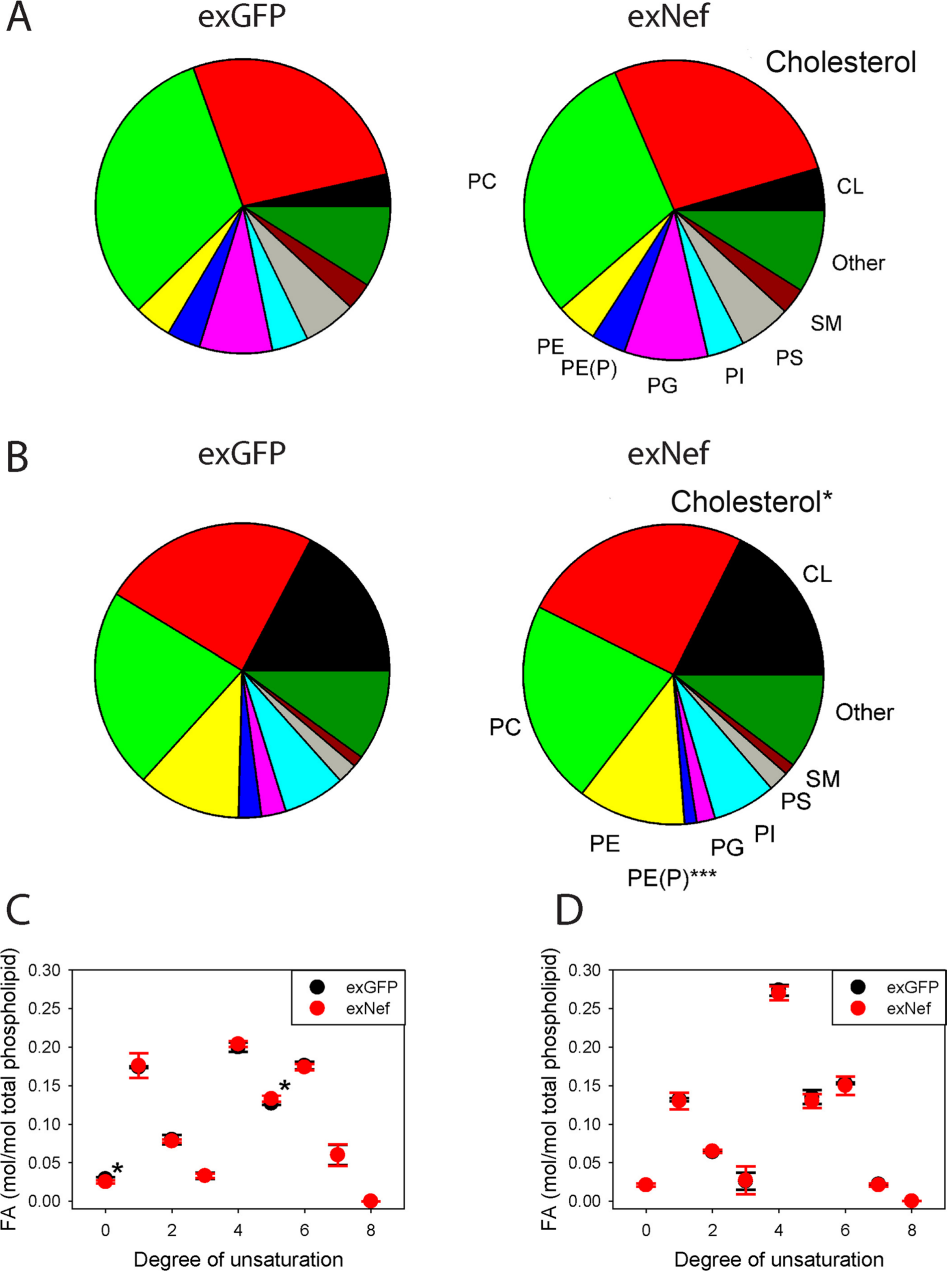
The effect of exNef on lipid raft lipidome in SH-SY5Y cells.**A**-**D** Lipids extracted from SH-SY5Y cells whole-cell lysate or isolated lipid rafts were analysed using liquid chromatography/mass-spectrometry (lipidomics) as described in “Methods”. All concentrations were normalized to the level of PC. A – The effect of exNef on relative abundance of the major lipid classes in lipid rafts; (*n*=3). **B** The effect of exNef on relative abundance of the major lipid classes in cell lysate; ***p<0.001, *p<0.05 versus exGFP (2-way ANOVA, *n*=3, biological replicates). **C** The effect of exNef on degree of saturation (number of double bonds) of fatty acids chains in raft lipids Means ± SEM , *p<0.05 versus exGFP (2-way ANOVA, *n*=3, biological replicates)). **D** The effect of exNef on degree of saturation (number of double bonds) of fatty acids chains in cell lysate, (*n*=3). *Abbreviations*: *CL, *Cardiolipin, *FA, *Fatty acids, *PC, *Phosphatidylcholine, *PE,* Phosphatidylethanolamine, *PE(P)*, Alkenyl phosphatidylethanolamine (plasmalogen), *P,* Phosphatidylglycerol, *PI,* Phosphatidylinositol, *PS,* Phosphatidylserine, *SM,* Sphingomyelin

To evaluate the impact of exNef on the lipid composition of neuronal lipid rafts, we performed lipidomics analysis of isolated raft-enriched PM fractions and corresponding whole-cell lysates. There was no effect of exNef on the abundance of either nine major (high-abundance) lipid classes (Fig. 2A) or minor (low-abundance) lipid classes (Supplemental spreadsheet). In the whole-cell lysate, however, exNef reduced abundance of plasmalogen (1.3 ± 0.1 vs. 2.6 ± 0.1%, *p* < 0001) and increased abundance of unesterified cholesterol (25.0 ± 0.3 vs. 23.8 ± 0.3%, *p* = 0.022) (Fig. 2B). Several low-abundance lipid classes were also altered by exNef treatment in whole-cell lysates (Supplemental spreadsheet).

A key factor governing lipid raft stability is the degree of saturation of the fatty acid chains. We observed only a marginal difference in the abundance of saturated fatty acids in lipid rafts between exGFP- and exNef-treated cells (0.029 ± 0.002 vs. 0.025 ± 0.002 mol/mol phospholipid, respectively; *p* = 0.049), accompanied by a slight reduction in polyunsaturated fatty acids containing five double bonds (0.13 ± 0.002 vs. 0.12 ± 0.004 mol/mol phospholipid, respectively; *p* = 0.037) (Fig. 2C). There was no effect of exNef on the degree of fatty acid saturation in the whole cell lysate (Fig. 2D). Thus, exNef exerted only limited effects on the lipid composition of neuronal lipid rafts.

When we compared the lipid composition of exGFP-treated lipid rafts with that of whole cell lysates, lipid rafts exhibited higher relative abundances of sphingomyelins (3.1 ± 0.1 vs. 1.3 ± 0.1%, *p* < 0.001), plasmalogen (3.8 ± 0.01 vs. 2.6 ± 0.1%; *p* < 0.001) and phosphatidylcholine (31.9 ± 1.5 vs. 21.7 ± 0.48%, *p* < 0.01) primarily at the expense of cardiolipin (3.3 ± 0.71 vs. 7.3 ± 0.5%, *p* < 0.001) and other phospholipids (Figs. 2A, B). Similar compositional differences were observed in exNef-treated cells when comparing lipid rafts to whole cell lysates (Figs. 2A, B).

### Physico-chemical properties of the lipid rafts

**Fig. 3:**
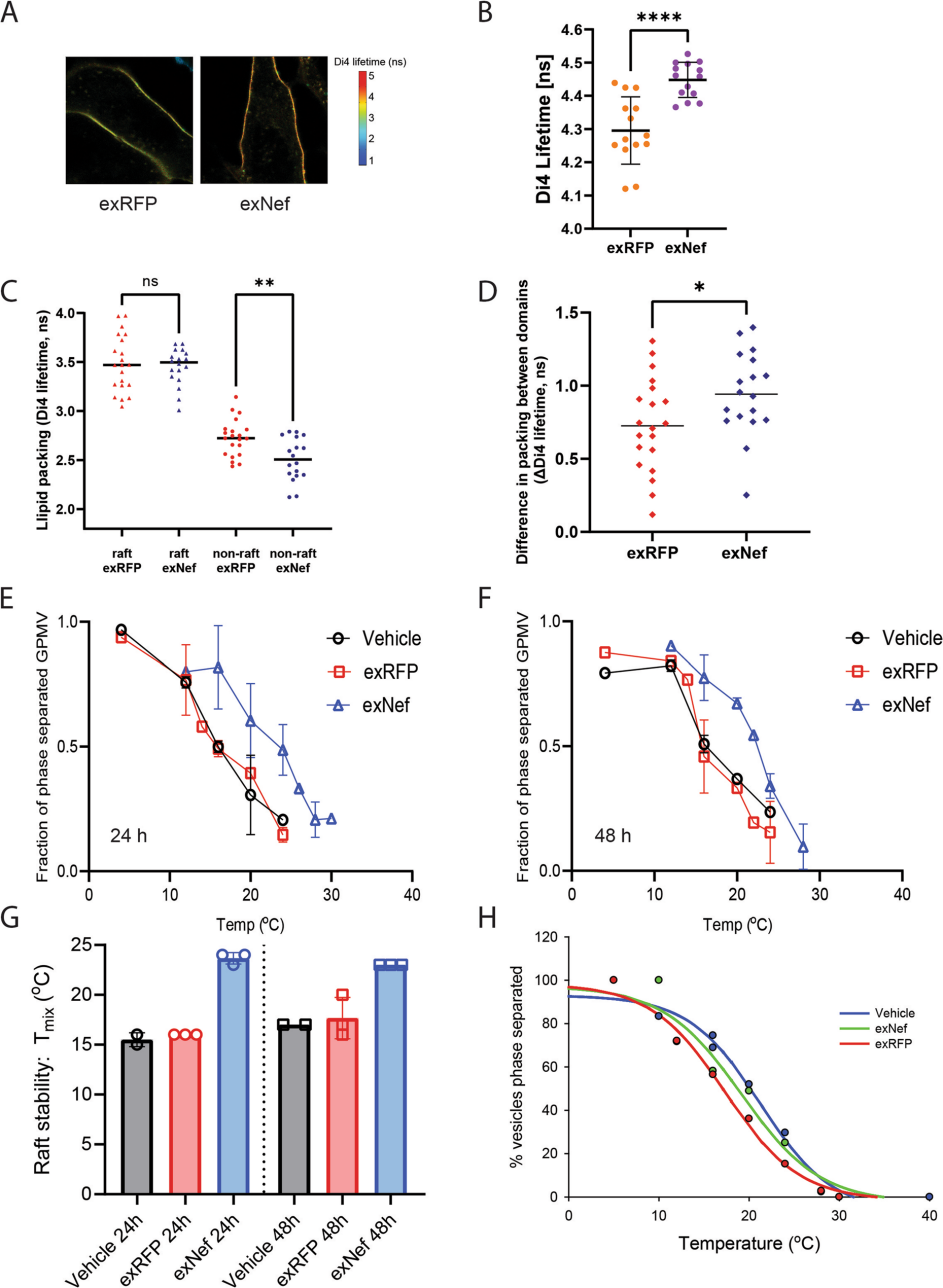
The effect of exNef on physico-chemical properties of the plasma membrane. **A **Representative FLIM images demonstrating the effect of exNef on Di4 lifetime in neuronal plasma membranes, which reflects their level of lipid packing. **B** Quantification of the effect of exNef on lipid packing in neurons measured by Di4 lifetime. **C **The effect of exNef on lipid packing in coexisting raft and non-raft domains in GPMV derived from THP-1 cells. **D** The difference in raft versus non-raft lipid packing in GPMVs is increased by exNef. **E **The effect of 24 h exNef treatment on raft phase separation temperature in GPMVs (*n* = 3, biological replicates). **F** The effect of 48 h exNef treatment on raft phase separation temperature in GPMVs (*n* = 3, biological replicates). **G** Quantification of exNef effect on lipid raft stability (i.e. phase separation temperature) in GPMVs. **H** The effect of exNef on lipid raft stability when treating isolated GPMVs rather than intact THP-1 cells. Vehicle refers to a buffer without any EVs. *p<0.05; **p<0.01, ****p<0.0001 versus exRFP treated cells (2-way ANOVA)

To assess whether exNef alters the physico-chemical properties of lipid rafts, we stained the plasma membranes of SH-SY5Y cells with Di4-ANEPPDHQ (Di4), a fluorescent stain whose emission lifetime is proportional to the degree of lipid packing in the membrane. Visual observation (Fig. 3A) and quantitation of the Di4 lifetime (Fig. 3B) indicate that treatment of SH-SY5Y cells with exNef results in a considerably tighter packed (i.e. more ordered) plasma membrane. The dynamic nature of lipid rafts in whole cells makes it challenging to determine which portion of the membrane, lipid raft, non-raft or both are affected by the treatment. This difficulty can be circumvented by creating giant plasma membrane vesicles (GPMV), where stable separation of raft and non-raft domains can be achieved. However, we were unable to produce GPMV from SH-SY5Y cells for reasons that remain unclear. Therefore, to assess the effects of exNef on physico-chemical properties of lipid rafts, we employed GPMVs derived from THP-1 cells. This substitution is justified because our previous studies demonstrated that the effects of exNef on cholesterol metabolism in THP-1 cells are similar to those in SH-SY5Y cells, indicating that THP-1 cells represent a valid surrogate system for these experiments [8, 15]. Unexpectedly, we found that lipid packing within the lipid raft fraction of the membrane was not affected by exNef, whereas packing of the non-raft fraction was less tight (Fig. 3C). However, the overall lipid packing was tighter after treatment with exNef (Fig. 3D). This finding appears paradoxical, as there was no increase in tightness of lipid packing of neither raft nor non-raft fractions, while the lipid packing of the whole PM membrane was tighter. The most likely explanation is that the overall increase in tightness of lipid packing in the PM is due to relative enrichment of lipid rafts compared with non-raft regions, rather than a change in the intrinsic properties of either domain. This interpretation is supported by our lipidomics analysis, which revealed no significant effect of exNef on lipid composition, a major determinant of raft physicochemical characteristics. Consistent with the increased packing difference between raft and non-raft phases (Fig. 3D), phase separation temperature in GPMV was increased following treatment of cells with exNef for 24 h (Fig. 3E) or for 48 h (Fig. 3F, summarized in Fig. 3G). These findings also show that exRFP does not affect packing when compared to vehicle (Figs. 3E-G). Finally, to determine whether exNef directly alters membrane properties, we isolated GPMVs and treated them directly with exNef, rather than applying exNef to intact cells. Under these conditions, exNef produced no detectable effect (Fig. 3H), indicating that its impact on membrane organization requires involvement of cellular machinery.

### Protein composition of the lipid rafts

**Fig. 4:**
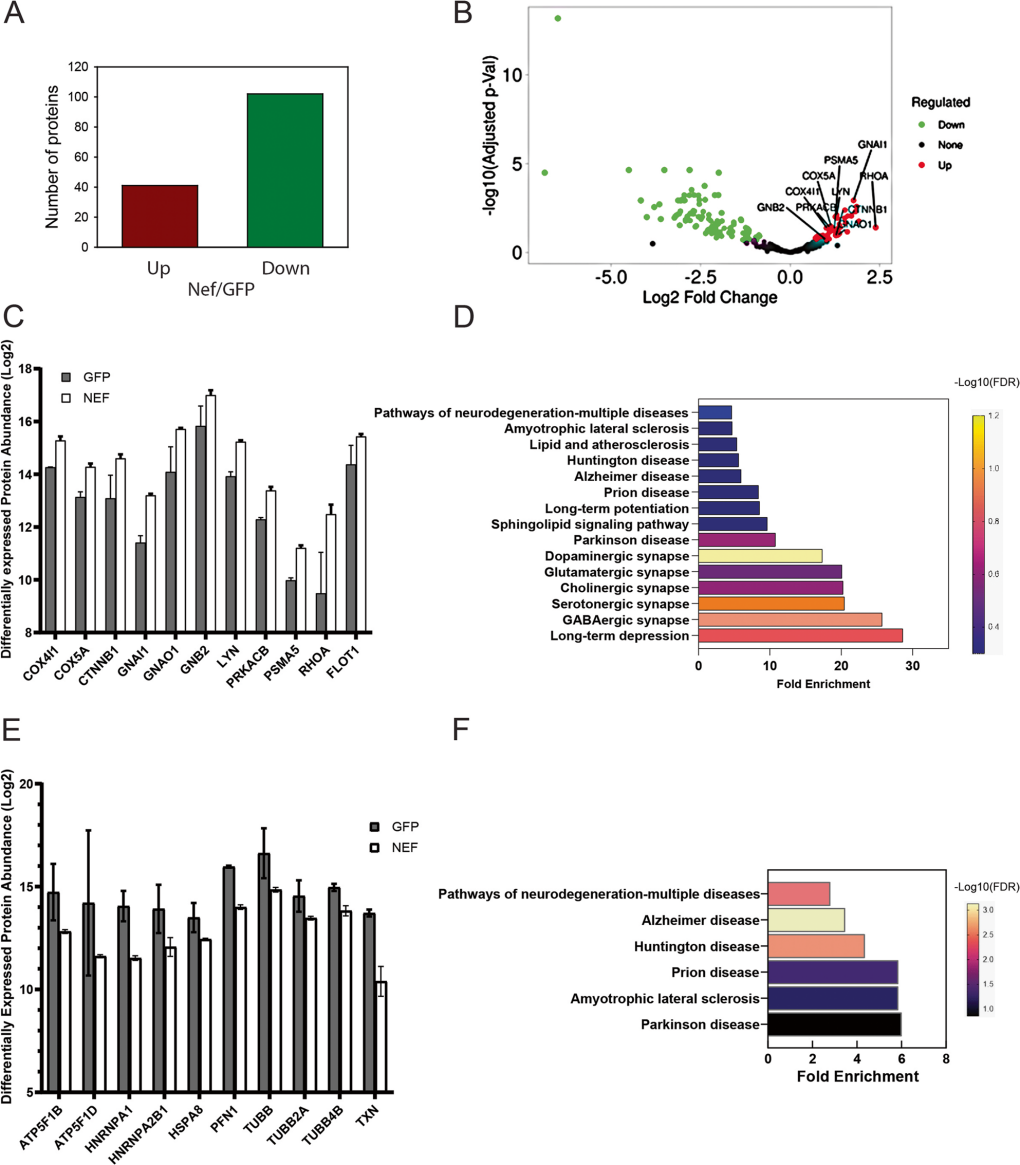
Unbiased proteomics analysis of the effect of exNef on protein composition of neuronal lipid rafts.** A ** Number of lipid raft proteins whose abundance was affected by exNef (exNef vs. exGFP); **B** Volcano plot of up- and down- regulated proteins; **C** Relative difference in the abundance of 10 proteins upregulated by exNef and involved in pathways of neurological disorders; (*n* = 3); **D** Pathways and diseases where the upregulated proteins are involved; **E** Relative difference in the abundance of 10 proteins downregulated by exNef and involved in pathways of neurological disorders; (*n* = 3); **F** Pathways and diseases where the downregulated proteins are involved

Two approaches were employed to investigate the effect of exNef on protein composition of the lipid rafts. First, we performed an unbiased proteomics analysis of lipid rafts isolated from SH-SY5Y cells treated with exNef. Because we observed substantial interexperimental variability in both the abundance and detection of low-abundance proteins, likely reflecting technical limitations of their measurement, we limited our analysis to high-abundance proteins, for which quantification was more reliable. These were defined as proteins consistently detected and reliably quantified across all experimental repeats and replicates. This filtering process yielded a final set of 296 candidate proteins. When we compared the list of these proteins with RaftProt database (https://raftprot.org/), 282 proteins (95%) were identified as human lipid raft proteins, confirming the quality of our preparation. The effect of exNef on the abundance of all 296 proteins was analysed using iDEP 2.0, with a false discovery rate (FDR) of 0.15. The abundance of 41 proteins was increased while the abundance of 102 proteins was reduced in lipid rafts from exNef-treated cells (Fig. 4A). Volcano plot depicting these effects is shown in Fig. 4B. A k-means search was conducted in the KEGG database, focusing on biological processes, cellular components, and molecular functions. This analysis revealed that 10 out of 41 upregulated proteins (Fig. 4C) were involved in 15 neurologically significant pathways and diseases (Fig. 4D). Two of the remaining 31 proteins, SPTBN1 [34] and GBA [35], were also found to be involved in a neurological pathology. Among 102 downregulated proteins we identified 10 proteins involved in pathways related to neurodegenerative conditions (Figs. 4E, F). Thus, unbiased proteomics analysis of lipid raft composition demonstrated that exNef regulated the abundance of a significant proportion of raft proteins implicated in pathogenesis of neurological disorders.

**Fig. 5:**
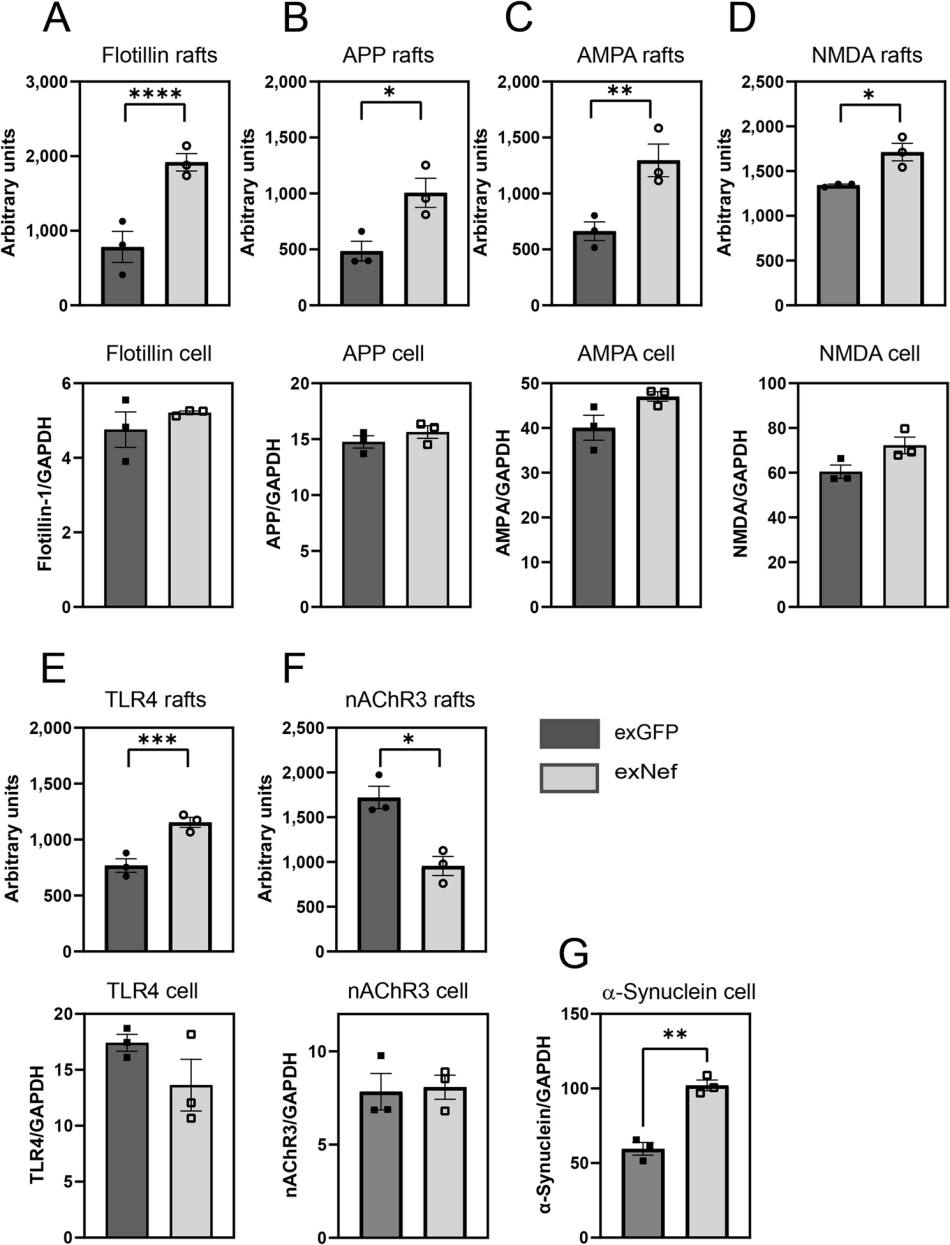
Targeted analysis of the effect of exNef on lipid raft abundance of proteins involved neurodegeneration and inflammation. Isolated lipid rafts (top row) or cell lysate (bottom row) of SH-SY5Y cells treated with exNef (or exGFP as controls) were subjected to PAGE and relative abundance was analysed by Western blot: **A** -Flotillin-1, **B **APP, **C **AMPA, **D ** NDMA, **E** TLR4, **F** - nAChR3, **G** - α-Synuclein. Means ± SEM; *p<0.05; **p<0.01, ***p<0.001 versus exGFP treated cells (2-way ANOVA, *n*=3, biological replicates).

To supplement the unbiased proteomics approach, we performed a targeted analysis of the effect of exNef on the abundance of several lipid raft proteins known to be involved in pathways of neurodegeneration and neuroinflammation. As expected, exNef elevated the abundance of the lipid raft marker flotillin-1 in rafts, but not in the cell lysate (Fig. 5A). Treatment of cells with exNef also increased the lipid raft abundance of several proteins playing key roles in neurodegeneration [36, 37]: amyloid precursor protein (APP) (Fig. 5B), α-amino-3-hydroxy-5-methyl-4-isoxazole propionic acid receptor (AMPA) (Fig. 5C) and N-methyl-D-aspartate receptor (NMDA) (Fig. 5D). The total abundance of these proteins in whole-cell lysates was unchanged by exNef treatment, consistent with their redistribution from non-raft regions to lipid rafts. The abundance of TLR4, a principal inflammatory receptor, was also selectively enriched in lipid rafts of exNef-treated cells (Fig. 5E), whereas the lipid raft abundance of the acetylcholine receptor nAChR3, a key regulator of ion channels, was selectively reduced (Fig. 5F). Thus, treatment of neurons with exNef altered lipid raft abundance of several proteins known to be involved in neurodegeneration and neuroinflammation. Finally, we assessed whether exNef alters cellular α-synuclein abundance, as α-synuclein is implicated in neurodegenerative pathology. Extracellular α-synuclein is taken up through transient interactions with lipid rafts [38], and intracellular α-synuclein biosynthesis is known to be regulated by raft abundance [39]. Consistent with this, treatment of cells with exNef resulted in increased α-synuclein levels in whole-cell lysate (Fig. 5G). Notably, α-synuclein was not detected in the lipid rafts.

### Functional consequences of exNef-induced changes in the lipid rafts

**Fig. 6:**
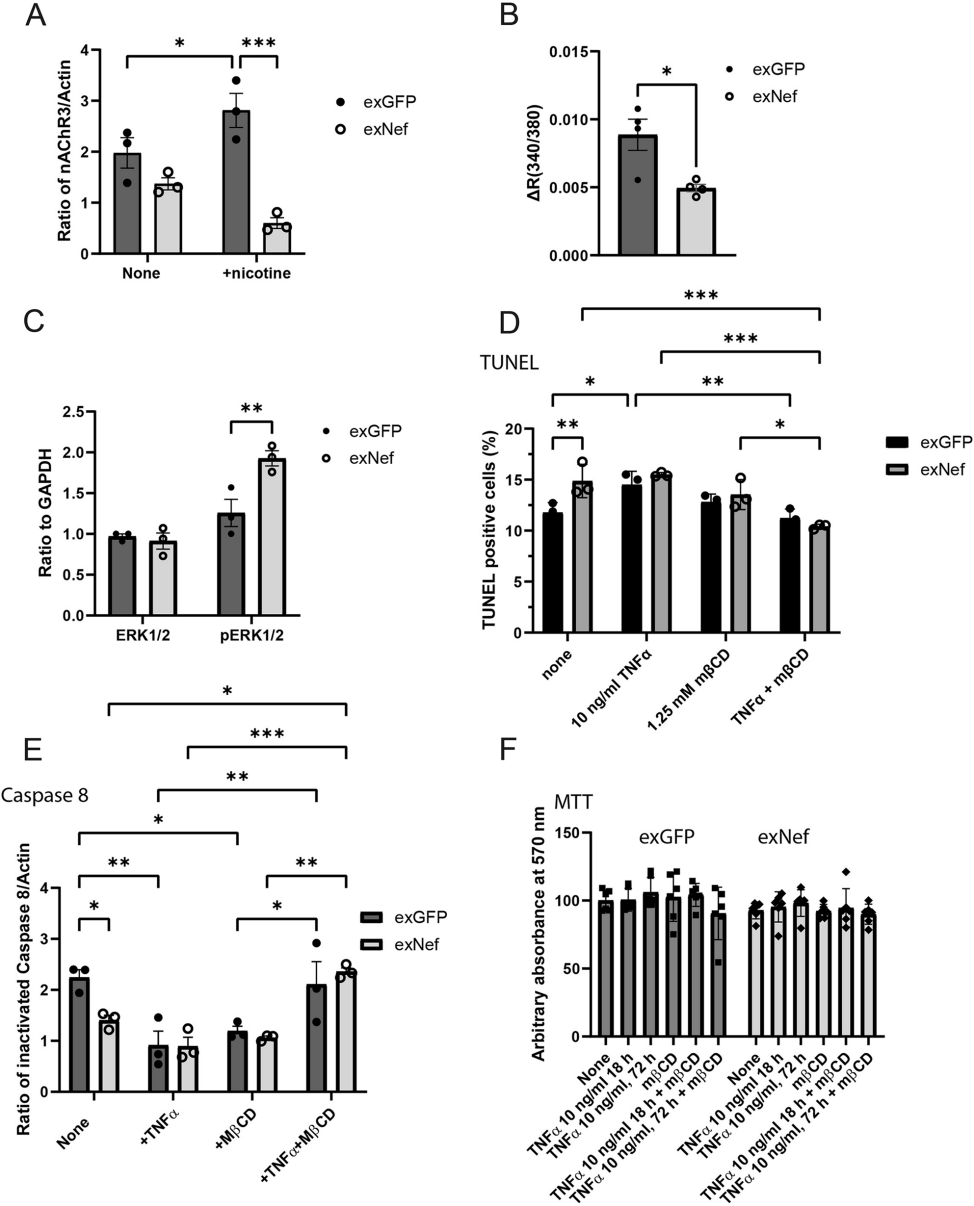
The effect of exNef on functional properties of lipid rafts.** A** Effect of exNef and nicotine (50 µM, 72 h) on the nAchR3 abundance in lipid rafts (quantitation of Western blots); **B** Effect of exNef on nAchR3 activity in response to activation with nicotine (50 µM, 72 h). NAchR3 activity assay is described in Methods; **C** Effect of exNef on phosphorylation of pERK1/2 in response to activation of TLR4 with LPS. Abundance of ERK1/2 and phosphorylated ERK1/2 was quantified by Western blot; **D** Effect of exNef and TNFα (10 ng/ml) on apoptosis (TUNEL assay); **E** The effect of exNef and TNFα on apoptosis (Caspase 8 assay); **F** The effect of exNef on cell viability (MTT assay). Means ± SEM; *p<0.05; **p<0.01, ***p<0.001 (2-way ANOVA, *n*=3, biological replicates)

To determine whether exNef-induced changes in lipid raft abundance and composition have functional consequences, we examined several raft-dependent signalling pathways in neurons. ExNef triggered the redistribution of the neurotransmitter receptor nAChR3 away from the lipid rafts (Fig. 5F), an effect that was further amplified following receptor activation with nicotine (Fig. 6A). This redistribution had functional implications: nicotine-induced activation of nAChR3 was significantly impaired in exNef-treated cells (Fig. 6B).

ExNef triggered re-localization of TLR4 to the lipid rafts (Fig. 5E). Lipid raft localization of TLR4 has been reported to be essential for its dimerization and activation [40], and we observed that exNef-induced re-localization was associated with increased phosphorylation of ERK1/2, a marker of TLR4 activation (Fig. 6C), similar to the previously reported effect of exNef on macrophages [15]. It must be noted that TLR4 is not the only receptor triggering phosphorylation of ERK1/2, and while the predominant role of this pathway in neuroinflammation and neurodegeneration was characterized for glial cell [41], contribution of neuronal TRL4 to these events is less clear.

Another essential element of neurodegeneration is apoptosis. Although exNef has been reported to trigger apoptosis in T cells [42], the underlying mechanisms remain unclear and may vary across cell types. Because TNFα is a potent inducer of apoptosis in neurons [43], and because effective TNFα signaling requires TNFR localization within lipid rafts [44], we asked whether exNef-induced alterations in raft composition might influence this pathway. To test this, we disrupted lipid rafts by depleting membrane cholesterol with methyl-β-cyclodextrin (MβCD). Both exNef and TNFα independently induced early apoptotic changes in neurons, and in both cases, disrupting lipid rafts prior to treatment attenuated these responses (Figs. 6D, E), consistent with the known raft dependence of TNFα signaling. However, exNef did not potentiate TNFα-induced apoptosis. Moreover, overall cell viability, as measured by the MTT assay, was unchanged across all treatment conditions (Fig. 6F), indicating that the observed effects did not progress to overt neuronal cell death under our experimental conditions. Together, these findings suggest that while TNFα-induced apoptosis is lipid-raft dependent, exNef-mediated raft remodeling alone is insufficient to amplify this apoptotic pathway in neurons.

### Reversal of exNef-induced lipid raft modifications

**Fig. 7:**
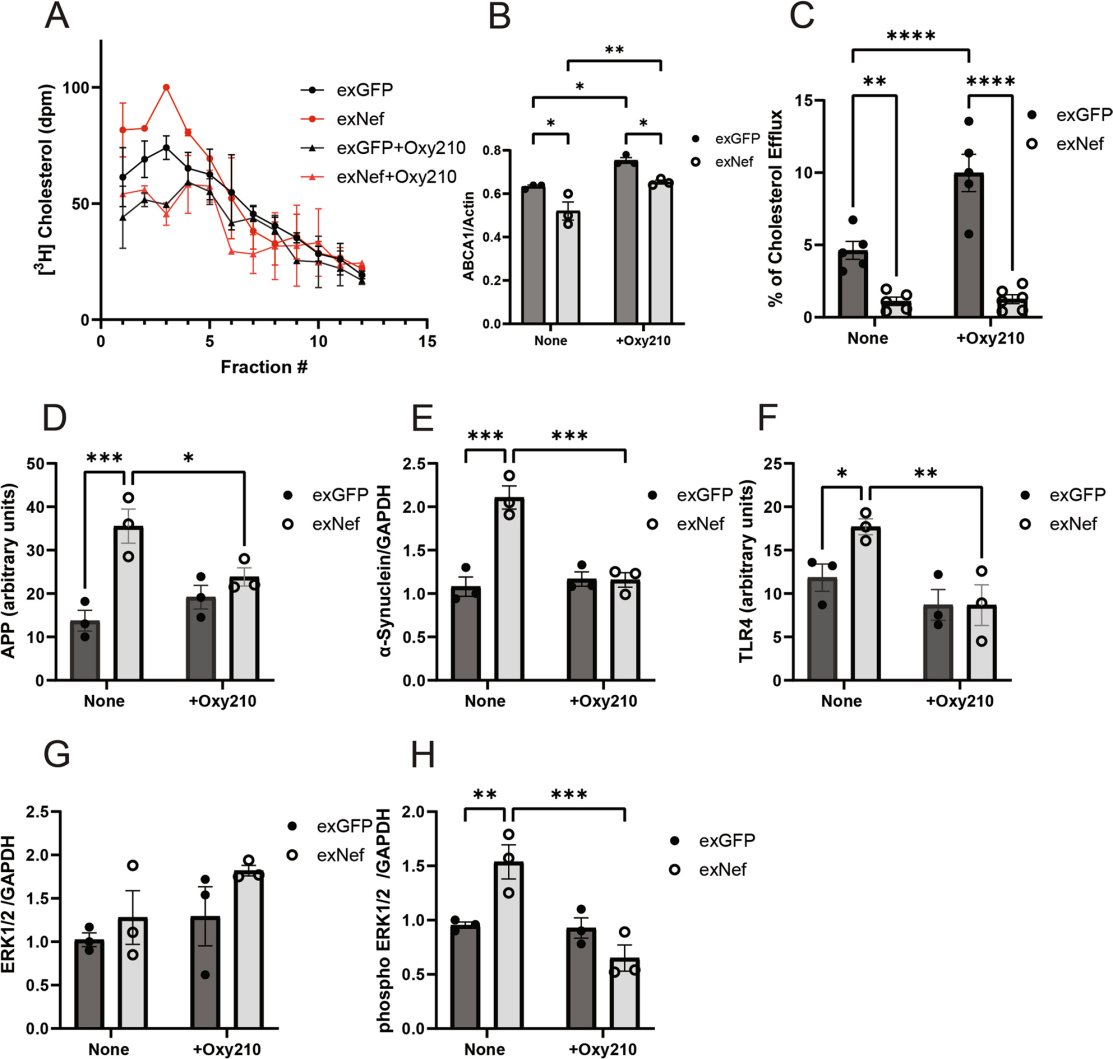
Reversal of the exNef-induced modification of lipid rafts. **A** The effect of exNef on the lipid raft abundance in the presence of Oxy210 (Means ± SD; p < 0.03 for exNef versus exNef+Oxy210; Mann-Whitney test; n = 3, biological replicates); **B** The effect of exNef on the ABCA1 abundance in the presence of Oxy210; **C** The effect of exNef on cholesterol efflux in the presence of Oxy210; **D** The effect of exNef on the APP abundance in lipid rafts in the presence of Oxy210; **E** The effect of exNef on the cellular abundance α-synuclein in the presence of Oxy210; **F** The effect of exNef on the TLR4 abundance in lipid rafts in the presence of Oxy210; **G** The effect of exNef on ERK1/2 abundance in the presence of Oxy210; **H** The effect of exNef on phosphorylated ERK1/2 abundance in the presence of Oxy210. **B**-**H**: Means ± SEM; *p<0.05; **p<0.01, ***p<0.001 (2-way ANOVA, *n*=3, biological replicates)

To determine whether lipid raft remodeling is essential for the functional effects of exNef, we tested whether these effects persist when exNef-induced raft elevation is alleviated. We used the compound Oxy210 (kindly provided by MAX BioPharma, Inc., Santa Monica, California, USA) to suppress the exNef-induced increase in the lipid raft abundance. Oxy210 is an anti-inflammatory oxysterol capable of graded reduction of lipid raft abundance in various cells including neurons [45, 46]. In SH-SY5Y cells, Oxy210 had a modest effect on lipid rafts in cells treated with exGFP, but reduced lipid rafts in exNef-treated cells to the level similar to that in exGFP-treated cells (Fig. 7A). The two most important cellular effects of exNef, reduction of ABCA1 abundance and cholesterol efflux [15], were preserved on the background of elevated ABCA1 abundance and cholesterol efflux caused by treatment with Oxy210 (Figs. 7B, C). Concentration of APP in lipid rafts was not affected by Oxy210 in cells treated with exGFP, however, Oxy210 mitigated exNef-induced elevation of APP abundance in lipid rafts (Fig. 7D). Similarly, Oxy210 mitigated exNef-mediated elevation of cellular α-synuclein abundance reducing it to the level found in exGFP-treated controls (Fig. 7E). Furthermore, Oxy210 effectively mitigated exNef-induced relocalization of TLR4 to the lipid rafts (Fig. 7F). While neither exNef nor Oxy210 affected cellular abundance of total ERK1/2 (Fig. 7G), exNef-induced phosphorylation of ERK1/2 was reduced by Oxy210 to the control level (Fig. 7H). Thus, reduction of lipid raft abundance by Oxy210 mitigated the functional effects of exNef.

#### Analysis of brain of PLWH diagnosed with HAND

To assess how these in vitro findings relate to HAND-associated pathology in the human brain, we compared exNef-induced alterations in the neuronal lipid raft proteome with transcriptomic changes observed in neurons from brains of people living with HIV (PLWH) diagnosed with HAND. Although direct comparison between the protein composition of a subcellular compartment in vitro and gene expression profiles from human brain tissue has inherent limitations, identifying convergent patterns across these datasets can highlight shared molecular pathways and mechanisms relevant to HAND pathogenesis. Accordingly, two comparisons were analyzed: lipid raft proteomes of cells treated with exNef versus exGFP, and differential gene expression in neurons from PLWH *versus* uninfected individuals.

**Fig. 8:**
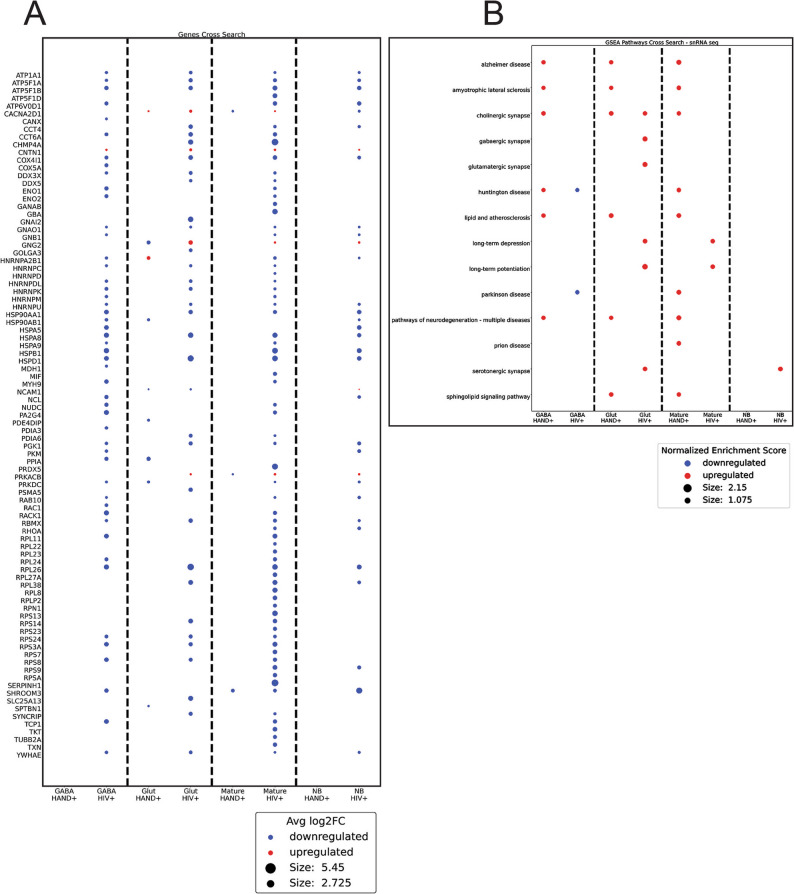
Comparison between the effect of exNef on protein composition of neuronal lipid rafts and the effects of HIV infection and HAND status on gene expression and pathway enrichment in human brain. **A** Wilcoxon Test of pertinent neuronal lipid rafts genes in the brain of PLWH. Changes of expression in PLWH relative to that in uninfected brain are shown in the column marked HIV + and changes of expression in PLWH HAND relative to that in brain of PLWH without HAND shown in the column marked HAND+. Only adjusted p-values of less than or equal to 0.05 were retained in the figure. **B** Gene Set Enrichment Analysis (GSEA) of the affected neuronal lipid rafts metabolic pathways between the brain of PLWH and uninfected brain (HIV+) and between PLWH with HAND and PLWH without HAND (HAND+). Only p-values of less than or equal to .05 were retained in the figure. *Abbreviations*: *GABA,* GABAergic neurons, *Glut,* *G*lutamatergic neurons, *NB,* Neuroblasts. *HAND+,* comparison of HAND+ to HAND- brains, *HIV+*, Comparison between PLWH (combined HAND+ and HAND- brains) and uninfected brains

The identity of the genes corresponding to the lipid raft proteins affected by exNef and those with elevated expression in the brains of PLWH as compared to uninfected individuals is shown in Fig. 8A; enriched metabolic pathways for these genes are shown in Fig. 8B. Remarkably, expression of almost all genes corresponding to proteins abundance of which in lipid rafts was affected by exNef was also affected by HIV infection in mature neurons and GABAergic neurons in human brain, with an expression of significant proportion of these genes also affected in glutamatergic neurons and neuroblasts (Fig. 8A). Eight out of 15 metabolic pathways identified as affected by exNef in the lipid raft proteomics study were found to be also affected by HIV infection in snRNA-seq analysis of the brain (Fig. 8B). Thus, there was a remarkable similarity between the effects of exNef on protein composition of lipid rafts in vitro and the effects of HIV infection on the neuronal expression of the individual genes and enrichment of metabolic pathways in the human brain. Although gene-level analysis of the snRNA-seq data did not reveal overlap between identities of the specific genes whose brain expression was altered in HAND and the individual raft proteins affected by exNef, pathway-level analysis revealed coordinated changes in the expression of these gene sets across nine pathways associated with neurological and neurodegenerative diseases (Figs. 8A, B). This pattern likely reflects a scenario in which subtle, individually non-significant gene expression changes converge to produce a detectable pathway-level signal. The notable similarity between the pathways affected by HIV infection in the human brain and those altered by exNef in neuronal lipid rafts in vitro suggests that the processes captured in our experimental system may mirror aspects of pathway-level dysregulation observed in PLWH, thereby supporting the potential relevance of these findings to brain biology.

## Discussion

In this study, we demonstrated that reorganization of neuronal lipid rafts by microglia-derived EVs containing the HIV protein Nef produces changes consistent with pathways implicated in neuroinflammation and neurodegeneration in the context of HAND. Neuronal lipid rafts play a prominent role in pathogenesis of neurodegenerative diseases [4]. Many amyloidogenic proteins accumulate in lipid rafts, and the rate, and even initiation of their misfolding, critically depends on this localization [47]. Inflammatory receptors, such as TLR4, also concentrate in the rafts where they undergo ligand-induced dimerization and subsequently initiate inflammatory signalling cascades [48]. Increased abundance and altered composition of lipid rafts may induce apoptosis by triggering apoptotic signalling [49–51]. Lipid rafts serve as key platforms for numerous neurotransmitter receptors and play a critical role in regulating neurotransmitter release [52]. Thus, Nef, an HIV protein responsible for modification of lipid rafts by the virus [15], may represent a major contributor to the pathogenesis of neurological disorders associated with HIV infection. Nef was detected in the brain tissue of some PLWH diagnosed with HAND, with concentrations reaching 0.13 ng/mg of total brain protein [8]. It is, however, essential to acknowledge that in addition to Nef, several other HIV proteins, such as gp120, Tat and Vpr, are secreted by HIV-infected cells [53, 54] and their contribution to the pathogenesis of neurological disorders associated with HIV infection cannot be ruled out.

Treatment with exNef did not alter the concentrations of cholesterol, sphingomyelins and plasmalogen in the lipid rafts, nor did it affect the saturation of fatty acid chains, a key determinant of raft stability. Accordingly, exNef did not affect the intrinsic stability of lipid raft domains. However, we observed tighter lipid packing in the isolated plasma membrane, most likely reflecting an increased proportion of raft domains. These findings indicate that exNef increases the abundance of neuronal lipid rafts without modifying their lipid composition or structural integrity. This is consistent with previously reported mechanisms by which exNef modulates raft abundance by targeting cytoskeleton, rather than by altering raft lipid composition [15].

While exNef had a minimal effect on raft lipid composition, it exerted a major effect on the protein composition of lipid rafts. Consistent with previous findings [8], exNef elevated amyloidogenic protein APP in the rafts, but not in the whole cell lysate, implying redistribution of this protein to lipid rafts. Similar changes were observed for AMPA and NMDA, two ionotropic glutamate receptors localized to lipid rafts [52], and TLR4, the key raft-associated inflammatory receptor [55]. In parallel, the abundance of the nicotinic acetylcholine receptor (nAChR3) in lipid rafts was reduced. This indicates differential enrichment or depletion of proteins in rafts. Changes in raft abundance of these proteins were accompanied by changes in their activity. Thus, phosphorylation of ERK1/2, a downstream effector of TLR4 activation, was increased, whereas activation of AChR3 was reduced. Furthermore, reversing the exNef-induced increase in lipid raft abundance with the synthetic raft antagonist Oxy210 also abolished the effects of exNef on ERK1/2 phosphorylation and on the accumulation of APP in lipid rafts or α-synuclein within the cells. ExNef-induced apoptosis was also raft-dependent; however, it was not contingent on TNFα, making it more likely to be caused by aggregation of β-amyloid, α-synuclein, or Tau. Collectively, these findings are consistent with a key role of lipid rafts in the multiple neuronal activities of exNef.

Remarkably, almost a quarter of high-abundance raft proteins found to be upregulated by exNef have a prominent function in pathways associated with neurological disorders, including neurodegenerative diseases, and/or lipid metabolism. Furthermore, when we compared our findings to the results of snRNA-seq analysis of brain samples of PLWH, all proteins and almost half of the pathways affected in our study were also found affected in neurons of PLWH. Although these two types of analysis reflect different levels of regulation in a different context, a remarkable level of similarity of the outcomes suggests that our findings reflect phenomena relevant to the processes in the brain of PLWH.

An intriguing question is what mechanism might underlie the exNef-induced redistribution of proteins to or away from lipid rafts. Because the lipid composition and stability of the rafts were unaffected by exNef, and the redistribution of proteins did not parallel changes in raft abundance, a passive mechanism appears unlikely. Although this possibility was not directly tested in the present study, one mechanism consistent with our findings could involve recycling endosomes. According to the model proposed by Balasubramanian et al. [56] and supported by subsequent studies [46, 57, 58], both recycled and newly synthesized plasma membrane components pass through a checkpoint in late endosomes during endocytic recycling. At this checkpoint, components of “disordered” plasma membrane regions are directed to lysosomes, whereas “ordered” ensembles are directed to the plasma membrane lipid rafts. The role of Nef in hijacking regulatory checkpoints within endocytic and secretory pathways is well established [11], and the late-endosomal sorting checkpoint governing trafficking into or away from lipid rafts represents a plausible point of interference for exNef. Several alternative explanations are consistent with existing literature, including: (i) Nef-mediated modulation of assembly of complexes of raft-associated proteins, which can reorganize surrounding lipids and thereby alter raft composition [59], (ii) Nef interference with post-translational protein modification and folding [13], which is known to influence lipid raft targeting, and (iii) altered competition among proteins for raft occupancy due to changes in trafficking dynamics or protein abundance.

### Limitations

The conclusions of this study are primarily based on in vitro experiments, and the direct relevance of these findings to neuronal processes in the brain of PLWH remains to be fully established. While this limitation was partially addressed by comparing affected pathways with transcriptomic data from PLWH brain samples, more definitive validation would require direct analysis of lipid raft protein composition in neurons isolated from human brain tissue. Additionally, due to technical challenges in isolating GPMVs from neurons, we used a different cell type to assess the physico-chemical effects of exNef on lipid rafts. Whether these effects are replicated in neurons remains to be determined.

## Conclusions

In conclusion, our findings indicate that treatment of human neurons with Nef-containing extracellular vesicles (EVs) released by microglial cells induces an increase in neuronal lipid raft abundance without altering their lipid composition. This structural remodelling was accompanied by the recruitment of multiple amyloidogenic and inflammatory factors into the lipid rafts, leading to enhanced inflammatory signalling and raft-associated apoptosis. Such reorganization of neuronal membrane microdomains may amplify local inflammatory responses and promote neurodegenerative processes characteristic of HAND. The strong concordance between transcriptomic alterations in neurons from HAND brains and proteomic changes observed in exNef-treated neuronal cells further supports the notion that Nef-mediated modulation of lipid rafts represents a mechanistic bridge linking viral persistence to neuronal dysfunction in HIV-associated neurocognitive disorder.

## Supplementary Information

Supplementary Material 1.

## Data Availability

All unique/stable reagents generated in this study are available from the lead contact, Dmitri Sviridov ( [Dmitri.Sviridov@Baker.edu.au](mailto:Dmitri.Sviridov@Baker.edu.au) ), with a completed materials transfer agreement. Any additional information required to reanalyse the data reported in this paper is available from the lead contact upon request. The datasets generated and/or analysed during the current study are available in the following repositories. The datasets of single-nucleus RNA-seq of the brain from human uninfected controls can be accessed from GEO (SRR19918318, SRR19918319, SRR19918320, SRR19918322, SRR19918323, SRR19918324, SRR19918325, SRR19918327). The datasets of single-nucleus RNA-seq of the four brain samples of PLWH can be downloaded from GEO (GSE296943). The mass spectrometry proteomics data have been deposited to the ProteomeXchange Consortium via the PRIDE partner repository with the dataset identifier PXD065713.

## Abbreviations

ABCA1: ATP binding cassette transporter type A1
AMPA: α-amino-3-hydroxy-5-methyl-4-isoxazole propionic acid receptor
APP: Amyloid precursor protein
BDNF: Brain-derived neurotrophic factor
Di4: Di4-ANEPPDHQ
EV: Extracellular vesicles
ERK: Extracellular Signal-Regulated Kinase
HAND: HIV-associated neurological disorders
HTHU: Highly transformed human microglial cells
ExGFP: GFP-containing extracellular vesicles
ExNef: Nef-containing extracellular vesicles
ExRFP: RFP-containing extracellular vesicles
GAPDH: Glyceraldehyde-3-Phosphate Dehydrogenase
GPMV: Giant plasma membrane vesicles
KRBB: Krebs-Ringer Bicarbonate Buffer
MβCD: Methyl-β-cyclodextrin
NMDA: N-methyl-D-aspartate receptor
nAChR3: Nicotine acetylcholine receptor 3
PM: Plasma membrane
PLWH: People living with HIV
TLR4: Tall-like receptor 4
TNFα: Tissue necrosis factor α
